# Now you see it, now you don't: flushing hosts prior to experimentation can predict their responses to brood parasitism

**DOI:** 10.1038/srep09060

**Published:** 2015-03-12

**Authors:** Daniel Hanley, Peter Samaš, Josef Heryán, Mark E. Hauber, Tomáš Grim

**Affiliations:** 1 Department of Zoology and Laboratory of Ornithology, Palacký University, 17. listopadu 50, Olomouc 77146, Czech Republic; 2 Department of Psychology, Hunter College and the Graduate Center, The City University of New York, 695 Park Avenue, New York, New York 10065, United States

## Abstract

Brood parasitic birds lay their eggs in other birds' nests, leaving hosts to raise their offspring. To understand parasite-host coevolutionary arms races, many studies have examined host responses to experimentally introduced eggs. However, attending parents often need to be flushed from their nests to add experimental eggs. If these birds witness parasitism events, they may recognize and reject foreign eggs more readily than parents who did not. We found that, after being flushed, female blackbirds, *Turdus merula*, remained close to their nests. Flushed females were more likely to eject foreign eggs and did so more quickly than females that were not flushed during experimentation. In contrast, flushing did not predict responses and latency to responses to parasitism by song thrush, *Turdus philomelos*, which flew farther from their nests and likely did not witness experimental parasitism. When statistically considering flushing, previously published conclusions regarding both species' response to experimental parasitism did not change. Nevertheless, we recommend that researchers record and statistically control for whether hosts were flushed prior to experimental parasitism. Our results have broad implications because more vigilant and/or bolder parents can gain more information about parasitism events and therefore have better chances of successfully defending against brood parasitism.

Some female birds avoid paying the high costs of parental care by laying their eggs parasitically in other birds' nests^1^. In response, host birds evolve fine-tuned recognition behaviours to reject foreign eggs from their nests. Discoveries from extensive research on avian brood parasitism have shaped biological thought, especially in the area of coevolutionary theory^2^,^3^,^4^. The vast majority of work, regardless of host, parasite, continent, or research team, has examined egg rejection through experimental parasitism^5^.

The standard protocol to test egg rejection abilities is to add a foreign egg into a host nest or manipulate a host egg(s), and record whether and how long it takes before the egg is rejected^6^,^7^. When adding experimental foreign eggs, birds may be attending their nests (e.g., incubating, shading, or guarding their eggs). To our knowledge, based on discussions with other researchers, the standard practice is to flush the bird from the nest, causing as little distress as possible, manipulate the nest content, and monitor the response of the host over a standardized period. Flushing is especially necessary during the incubation period when parent(s) are on their nests regularly (see Results).

Surprisingly, there has been no published research on whether hosts witnessing the experimental manipulation are more likely to respond to foreign eggs. Hosts that encounter adult parasites, or their experimental dummies, near their nests can increase egg rejection rates^6^. Disturbances near the nest whether from natural cuckoos^8^, experimental cuckoo or cowbird dummies^6^,^9^, or the researchers themselves^10^, draw the attention of nest owners, which may increase nest inspection behaviours and egg ejection^11^. Therefore it stands to reason that a host's egg discrimination could be facilitated when hosts watch the researcher place an egg within their nest.

Here we examine if flushing from the nest during artificial parasitism (hereafter, flushing) is a potential confound for antiparasitic responses in blackbirds, *Turdus merula* and song thrush, *T. philomelos.* We studied the egg rejection responses of these species to artificial, non-mimetic blue egg models^12^ (Figure 1) both in their native ranges in the Czech Republic (CZ), and in their introduced ranges in New Zealand (NZ). First we determined whether blackbirds and song thrush were equally likely to remain close-by as foreign egg models were added to their nests, by measuring how far females flew from their nests after flushing (hereafter, fleeing distance *sensu*^13^). Second, we determined if hosts' responses to experimental parasitism can be predicted by whether they were flushed from their nest at the time of experimental manipulation. Third, we assess whether this potential confound would change the previous conclusions^14^. If flushing predicts host response, our results will have broad implications for the design and interpretation of studies of brood parasite-host coevolution.

## Results

### Fleeing distance

When flushed from their nests blackbirds remained closer (median = 5 m, inter-quartile range = 8 m, *n = * 68 nests) and song thrush flew farther from their nests (median = 22.5 m, inter-quartile range = 19.5 m, *n = * 6; Mann-Whitney test: U = 75; *p = * 0.01). The blackbirds and song thrush included in this analysis had similar clutch sizes (blackbirds: median = 4 eggs, inter-quartile range = 2 eggs, *n* = 68; song thrush: median = 4 eggs, inter-quartile range = 0.75 eggs, *n* = 6; U = 265, *p* = 0.20) and were sampled at similar dates within breeding season (1 = 1^st^ January; blackbirds: median = 119, inter-quartile range = 37, *n* = 68; song thrush: median = 109, inter-quartile range = 47, *n* = 6; U = 214, *p* = 0.85). Although these data are from CZ, the fleeing distances appear consistent with our observations (PS) from NZ.

### Does flushing influence host responses?

**Figure 2:**
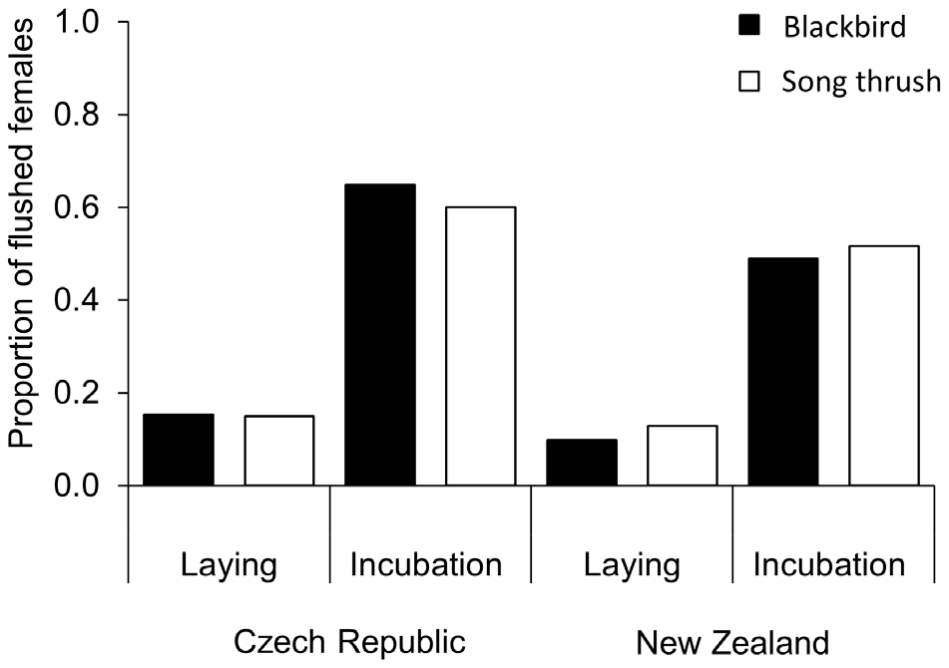
The proportion of female blackbirds (*n = * 242 in Czech Republic and *n = * 51 in New Zealand) and song thrush (*n = * 20 in Czech Republic and *n = * 31 in New Zealand) that were flushed from their nest were similar both during laying or incubation.

**Figure 3:**
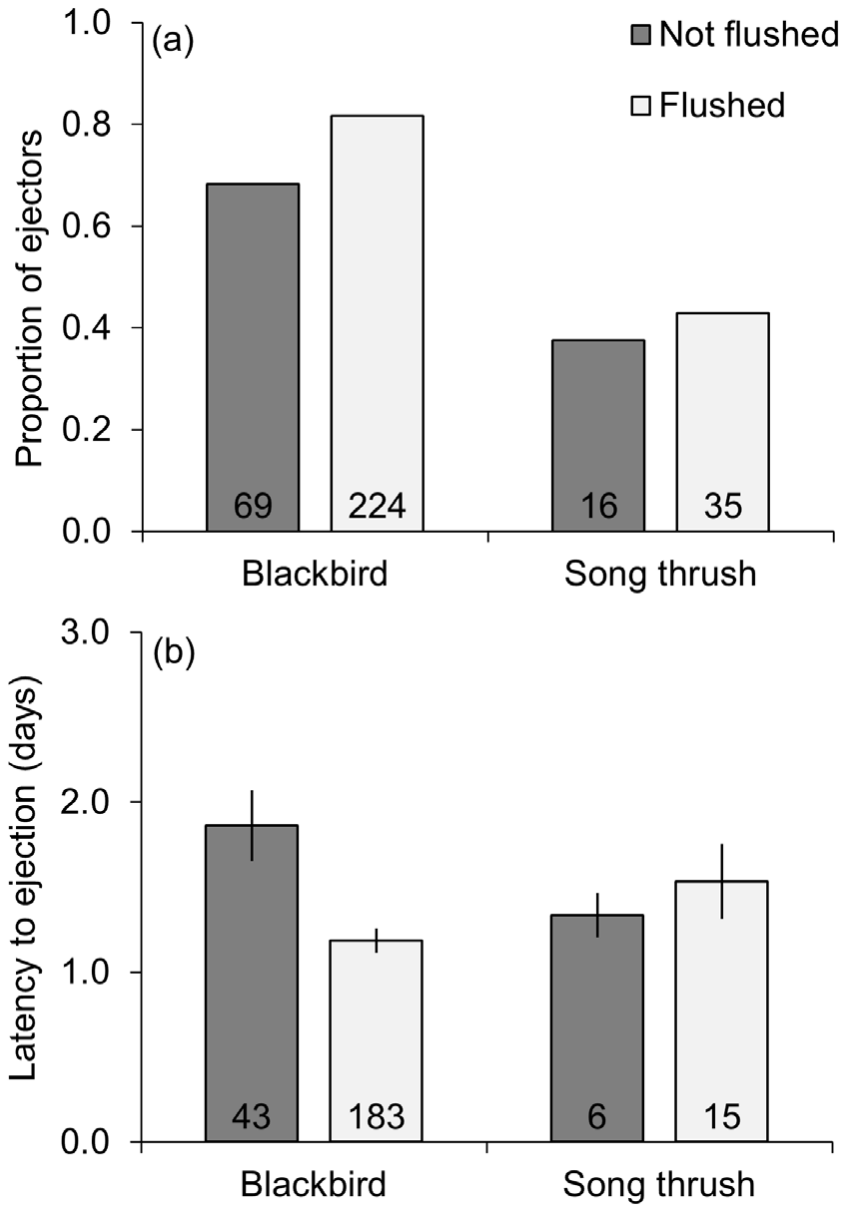
Ejections (a) of non-mimetic blue egg models were more common in flushed female blackbirds than those that were not flushed, but this was not the case for female song thrush. Latency to ejection (mean ± SE; b) was shorter in flushed blackbirds than blackbirds that were not flushed, while non-significantly longer in flushed than non-flushed song thrush.

The proportions of blackbirds (*n = * 293) and song thrush (*n = * 51) that were flushed were virtually identical for both species, across both laying and incubation stages (Figure 2). However, flushed blackbirds were much more likely to eject the non-mimetic blue egg model than blackbirds that were not flushed (OR = 2.7, CI_0.95_ = 1.4 to 5.1, *p = * 0.002; Table 1, Figure 3a), while flushing did not predict song thrush responses (OR = 1.2, CI_0.95_ = 0.32 to 5.2; *p = * 0.77; Table 1; Figure 3a). Similarly, flushed blackbirds ejected the models quicker than blackbirds that were not flushed, while the difference was in the opposite direction and non-significant in song thrush (Table 1; Figure 3b). Results of full and reduced models led to the same conclusions (Table 1).

### Reanalysis of published data

We found that flushing was also significant positive predictor of blackbird egg ejection in reanalyses of previously published data^14^ (Table 2), but not in the song thrush (Table 3). Blackbirds flushed before an experimental manipulation ejected 81% of the non-mimetic blue egg models, while females that were not flushed ejected only 60% of these egg models. Nonetheless, the inclusion of flushing did not change the previous conclusion that rejection rates and latencies to rejection of non-mimetic blue egg models did not covary geographically with risk of interspecific parasitism^14^. Flushing was a marginally non-significant predictor of the latency to ejection in the blackbird (Table 2) and a non-significant predictor in the song thrush (Table 3).

## Discussion

We found that blackbirds generally remained within 10 m of the nest after flushing and therefore may be able to routinely witness the experimental insertion of the foreign egg. Witnessing the addition of a foreign egg may increase the accuracy of parasitism detection. Accordingly, blackbird egg ejection responses and latency to ejection of non-mimetic blue eggs were best explained by flushing. These results suggest that blackbirds respond to witnessing parasitism events. In contrast, song thrush flew farther from their nests and were unlikely to witness the experimental manipulation. Indeed, flushing did not predict song thrush responses or latency to ejection. Our findings are congruous with the growing evidence that birds respond by altering their behaviours following encounters with researchers^15^,^16^. Thus, interspecific comparative studies may be biased because more vigilant species may show intrinsically higher ejection rates and shorter latencies to ejection.

Flushing may be an important confound of egg rejection in some species, and it was not previously considered and therefore raises concerns about the validity of prior research. However, we illustrate that while flushing is a statistically important predictor of host responses in blackbirds, it does not necessarily affect the interpretation of previously published results. It still remains to be assessed whether *Turdus* thrushes' tendencies to accept mimetic vs. reject non-mimetic foreign eggs^17^ may be influenced or confounded by whether the female was flushed or not. In addition, during natural parasitism events, just like in artificial parasitism events, hosts may or may not be present at their nests^8^ and flushing may even be used by parasites to discover host nest locations^18^,^19^. Therefore host responses to flushing may be adaptive, for example, if a host monitors the nest after it was flushed. Similar to our findings, the presence of a real or model adult parasite near the nest is a strong predictor of egg rejection behaviours in several host species^20^,^21^. Thus, our findings do not suggest that flushing during nest manipulation is a fatal flaw of previous work, but they do caution future brood parasitism research to consider whether and how hosts could use information about the experimental parasitism event to fine-tune their rejection responses.

It is possible that we were unaware of the presence of some females who witnessed the introduction of the egg model without being flushed from the nest (i.e., if they were silently hidden in the nearby vegetation before the observer approached the nest). Nonetheless, the strong statistical effects of flushing argue against this, or at least suggest that such witnessing produces a different effect. We were unable to determine if flushing *per se* or witnessing experimental parasitism was acting as a stimulus for egg rejection. But, in support of the witnessing parasitism scenario, we found that the interspecific differences in fleeing distances were consistent with interspecific differences in whether flushing explained host responses: blackbirds remaining close to the nest showed a positive effect of flushing on egg rejection rate and shorter latencies to ejection, whereas song thrush did not. These interspecific differences suggest that birds remaining close to the nest have a cognitive advantage over those that fly far away. Therefore we encourage future intraspecific experimentation to provide more insight into this emerging field of study.

Our findings suggest a need to modify classic field methods used in brood parasitism research. We recommend that researchers statistically control for whether they flushed a parent from the nest during experimental manipulation (categorical variable: “flushed” or “not flushed”), or only add foreign eggs when the location of the host(s) is known (e.g., radio telemetry, resighting, etc.). This is particularly true for bolder species that more aggressively defend their nests^22^ and for species where both sexes closely attend the nest and reject parasitic eggs^23^, and therefore parents could more often witness experimentation. Modern video surveillance and telemetry techniques provide a variety of tools to monitor nests prior to experimentation, allowing researchers some control over when experimental manipulations should occur.

There are many potential biological (e.g., predators, parasites, or researchers) and abiotic (e.g., wind or noise) disturbances that can flush parents from their nests. Any of these disturbances should induce birds, hosts or not, to check nest contents; however, only hosts of brood parasites, whether hetero- or conspecific, should perceive altered risk of parasitism and consequently adjust their response. The mechanism behind this effect, direct observation or flushing *per se*, remains to be determined. However, the main message of this study is a methodological one: no matter what the mechanism, the logistically inescapable effect of flushing may influence hosts' responses to experimental brood parasitism in general, and future research should take this into account.

## Methods

### General

We studied native *Turdus* thrush populations in Olomouc, Czech Republic (49°35'8″N, 17°15'3″E) in 2009–2014 and introduced thrush populations in Auckland, New Zealand (36°50′26″S, 174°44′24″E) in 2009^14^,^24^. Blackbirds and song thrush show very similar nest placement and habitat selection, including our study sites in both Czech Republic^25^,^26^ and New Zealand (own unpublished data from several studies^12^,^14^,^27^,^28^,^29^). Specifically, in all populations we sampled nests in similar habitats, from conifers and dense shrubs situated in public parks and gardens where nests of both species were interspersed. *Turdus* thrushes in Europe only rarely raise common cuckoo (*Cuculus canorus*) chicks successfully^12^,^30^,^31^. Current evidence suggests that blackbirds are conspecific parasites^14^.

Despite extensive and long-term mist-netting and colour-banding effort^32^, the exact identity of each tested female (only females eject in our study population^27^) was not always known. Therefore, we avoided sampling the same location after experimentation to reduce the chance of testing the same individuals more than once. However, prior experience with experimentation would not necessarily cause females to be more or less likely to flush on subsequent visits, and empirical data from the same egg model type showed that prior experience had only weak and statistically non-significant effect on ejection probability^27^.

We conducted this research in accordance with the Association of Animal Behaviour and the Animal Behavior Society guidelines for the treatment of animals in research. In the Czech Republic our research methods and protocols were approved by the Research Ethics Committee of Palacký University (45979/2001–1020), and the research was conducted under licenses from the Department of Environment of the City of Olomouc (SmOl/ZP/55/6181 b/2009/Pr and SMOVZP/55/8542/2011/Kol). Although no specific permissions were required to study these two model invasive species in New Zealand, this research was approved by the University of Auckland's Animal Ethics Committee (AEC/09/2006/R512).

### Fleeing distance

In 2014, in Olomouc, JH quantified how far female blackbirds and song thrush flew from their nests after flushing (to the nearest meter, up to 30 meters). Specifically, JH slowly walked to the nest until the female left, or, if necessary, he slowly moved a hand or mirror toward the nest. Then, JH estimated the average distance between the female and the nest between 10 and 20 s *after* flushing. This corresponds to when experimental parasitism events occurred (see below for details). We refer to this distance as fleeing distance. This should not to be confused with flight initiation distance^33^, which measures the distance between an observer and animal *before* the animal flees. We tested whether fleeing distance, manipulation dates within season (1 = 1^st^ January), and clutch sizes, differed between blackbirds and song thrush using Mann-Whitney U-tests. Levene's test detected no heterogeneity of variance for these variables.

### Does flushing influence host responses?

Nests were monitored for six days after introducing a single non-mimetic blue model egg, and models present or missing after this period were deemed accepted or ejected, respectively^14^,^24^. The mass, dimensions, and spectral reflectance^14^,^28^ of these egg models closely match the cuckoo eggs that are naturally found within common redstart *Phoenicurus phoenicurus* nests^34^. We used these non-mimetic blue models, because this is the most common egg model type used across Europe and has been used in the majority of studies on these species^27^,^28^,^35^. Using the same model type was necessary for making meaningful and quantitative comparisons of host behavior between species and populations^14^,^36^. Desertion was not a response to artificial parasitism^14^,^24^. For each experiment we recorded whether the female was flushed from the nest cup or not when the egg model was introduced. For both species we included only nests with final clutch sizes of 4–5 eggs in CZ and 3–4 eggs in NZ, which are typical in these populations^29^.

We ran separate statistical models for blackbirds and song thrush. We used Fisher Exact tests to determine if flushing related to host response to the model egg and present the associated odds ratios (OR) and confidence intervals (CI). We used generalized linear models to examine if flushing (yes or no) predicted host responses (binomial distribution) and latency to ejection (Poisson distribution). We controlled for other relevant variables^14^,^24^,^29^: year (categorical), the laying date of the first egg (continuous; 1 = 1^st^ April in CZ and 1 = 1^st^ September in NZ), geography (categorical; CZ or NZ), nest age (continuous; days), and clutch size (continuous). Laying date was centred for each year and geographic location (CZ or NZ) separately to remove potentially confounding effects of annual and seasonal variation^14^,^24^,^29^. We used Nagelkerke's R^2^ to estimate model fit^37^ for models with binomial responses and the difference between the null deviance and residual deviance divided by the null deviance (hereafter, pseudo R^2^) for the models with Poisson error distributions^38^. We performed model selection through backward elimination of non-significant terms. All of these analyses were conducted in R version 3.1.1.

### Reanalysis of published data

To examine whether flushing could have confounded previously published analyses and conclusions, we reanalysed our own previously published results using the same statistical approach^14^ (i.e. excluding flushing), and also including flushing as an additional predictor for both the blackbird and the song thrush. In these reanalyses, we used only data for the same non-mimetic blue egg models, because we did not record flushing data for other egg model types.

Our primary interest in these reanalyses was in the effects of flushing (binary predictor) and geography (categorical 3-level predictor: sympatry, micro-allopatry and macro-allopatry with the common cuckoo *Cuculus canorus*, a rare interspecific brood parasite of *Turdus* thrushes in Europe but not in New Zealand, for definitions see Ref. 14) on egg rejection (binary response variable). In addition to flushing and geography (see above), all statistical models included nest stage (categorical predictor with four levels: egg laying, 1–3 days of incubation, 4–9 days of incubation, 10 days of incubation to hatching), laying date (first egg laid; continuous), clutch size (clutch size at clutch completion; continuous). Laying date was centred (see above) within each year for CZ and NZ separately to remove confounding effects of between-year variation of seasonal breeding and timing of experiments. We did not include egg model and its interaction with geography as in the previous analyses^14^ because only responses to blue models were included (see above). Otherwise, we controlled for all variables that were previously included in these models^14^.

We selected final models by backward elimination of non-significant terms, retaining two main factors of interest (geography and flushing) in the model regardless their significance, following the previously published methods^12^,^39^. For consistency with previously published work, we report test statistics and P-values for non-significant terms from backward elimination procedure just before the particular term was removed from the model. These reanalyses were conducted in SAS version 9.2.

## Author Contributions

D.H. and P.S. collected the data on egg rejection, and J.H. collected data on fleeing distances. D.H. ran the analyses for fleeing distance and the influence of flushing on host responses, while P.S. reanalysed previously published data. D.H., P.S., M.E.H. and T.G. wrote the main manuscript text. All authors finalized the manuscript.

## Figures and Tables

**Figure 1:**
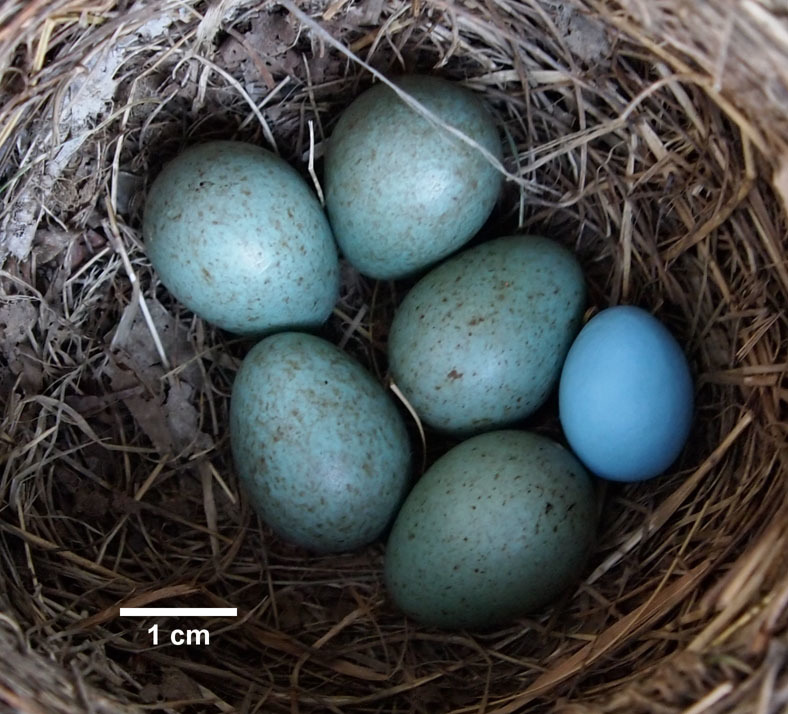
We introduced a single blue non-mimetic model into blackbird (depicted here, photograph taken by D. H.) and song thrush nests in their native European and introduced New Zealand ranges.

**Table 1:** Generalized linear model outputs predicting the behavioural response to experimental parasitism with a non-mimetic blue egg model (either egg ejection or acceptance) and its latency (for egg ejections only) for the blackbird and song thrush. We present full models and final reduced models (sequential backward elimination of non-significant terms) as well as the parameter estimates and a measure of standardized effect (z-score) to evaluate the direction and relative strength of each predictor. Significant predictors are in bold. D = dispersion with associated tests for over and under dispersion tests. See Materials and Methods in the main text for other details

		Full model					Reduced model				
Parameter		Estimate	SE	z	LR χ_1_^2^	*p*	Estimate	SE	z	LR χ_1_^2^	*p*
**Blackbird response** (R^2^ = 0.12, *n* = 293)							(R^2^ = 0.12)				
(Intercept)		0.12	1.42	0.08		0.94	0.07	0.30	0.22		0.83
Flushing		**0.92**	**0.34**	**2.73**	**7.32**	**0.007**	**0.91**	**0.31**	**2.91**	**8.28**	**0.004**
Year					**10.14**	**0.02**				**8.69**	**0.03**
Laying date		−0.0005	0.01	−0.08	0.01	0.94					
Geography†		0.54	0.56	0.96	0.93	0.33					
Nest age		0.06	0.04	1.47	2.21	0.14					
Clutch size		−0.12	0.31	−0.40	0.16	0.69					
**Song thrush response** (R^2^ = 0.26, *n* = 51)							(R^2^ = 0.16)				
(Intercept)		−1.73	3.57	−0.48		0.63	−0.43	0.31	−1.38		0.168
Flushing		0.18	0.71	0.25	0.06	0.80					
Year					0.61	0.44					
Laying date		**−0.03**	**0.02**	**−2.24**	**6.35**	**0.03**	**−0.03**	**0.01**	**−2.33**	**6.65**	**0.020**
Geography†		−0.65	1.08	−0.60	0.36	0.55					
Nest age		0.12	0.10	1.22	1.55	0.22					
Clutch size		0.34	0.74	0.46	0.21	0.64					
**Blackbird latency to ejection** (pseudo R^2^ = 0.10, *n* = 226, D = 1.05, *p* = 0.63)						(pseudo R^2^ = 0.04, D = 1.12, *p* = 0.32)					
(Intercept)		**1.58**	**0.57**	**2.79**		**0.005**	**0.62**	**0.11**	**5.55**		**<0.0001**
Flushing		**−0.46**	**0.14**	**−3.31**	**10.33**	**0.001**	**−0.45**	**0.13**	**−3.44**	**11.01**	**0.001**
Year					4.62	0.20					
Laying date		−0.003	0.002	−1.10	1.22	0.27					
Geography†		**−0.65**	**0.25**	**−2.60**	**6.85**	**0.009**					
Nest age		**−0.03**	**0.02**	**−2.29**	**5.31**	**0.02**					
Clutch size		−0.14	0.13	−1.11	1.26	0.26					
**Song thrush latency to ejection** (pseudo R^2^ = 0.25, *n* = 21, D = 0.52, *p* = 0.003*)							(pseudo R^2^ < 0.01, D = 0.81, *p* = 0.73)				
(Intercept)		2.07	2.13	0.97		0.33	**0.39**	**0.18**	**2.17**		**0.03**
Flushing		0.12	0.42	0.27	0.07	0.78					
Year					0.91	0.34					
Laying date		−0.001	0.01	−0.05	0.002	0.96					
Geography†		−0.73	0.59	−1.23	1.46	0.23					
Nest age		−0.05	0.05	−0.89	0.84	0.37					
Clutch size		−0.28	0.44	−0.63	0.38	0.53					

*Due to evidence of underdispersion we examined other distributions: negative binomial, quasi-Poisson, and Gaussian. In every case our results were quantitatively similar and qualitatively (i.e., as for conclusions) identical to those presented here.

^†^*The effect of geography was calculated with reference to CZ.*

**Table 2:** Egg rejection response and latency to rejection by blackbirds. Test statistics for predictors of blackbird response and latency ejection just prior to elimination for models including flushing as a predictor and models not considering flushing as a predictor. Significant terms from final models are in bold. Egg ejection was elicited by placing an artificial non-mimetic blue egg model into each nest

	Including flushing			Excluding flushing		
	ddf	F	*p*	ddf	F	*p*
**Ejection**						
Flushing	**272**	**6.68**	**0.01**	-	-	-
Geography	**272**	**3.83**	**0.02**	**273**	**5.67**	**0.004**
Nest stage	269	1.61	0.19	270	2.54	0.06
Laying date	264	0.19	0.66	269	0.62	0.43
Clutch	265	1.32	0.25	265	0.99	0.32
**Latency to ejection**						
Flushing	208	3.08	0.08	-	-	-
Geography	208	2.15	0.12	209	2.54	0.08
Nest stage	**208**	**14.10**	**0.0002**	**209**	**16.28**	**<0.0001**
Laying date	207	0.70	0.40	208	0.90	0.34
Clutch	203	0.26	0.61	204	0.33	0.57

**Table 3:** Egg rejection response and latency to rejection by song thrush. Test statistics for predictors of song thrush response and latency ejection just prior to elimination for models including flushing as a predictor and models not considering flushing as a predictor. Significant terms from final models are in bold. Egg ejection was elicited by placing an artificial, non-mimetic, blue egg model into each nest

	Including flushing			Excluding flushing		
	ddf	F	*p*	ddf	F	*p*
**Ejection**						
Flushing	52	0.69	0.41	-	-	-
Geography	52	1.56	0.22	53	1.75	0.18
Nest stage	47	0.18	0.91	48	0.09	0.97
Laying date	**52**	**5.41**	**0.02**	**53**	**5.33**	**0.02**
Clutch	50	0.58	0.45	51	0.70	0.41
**Latency to ejection**						
Flushing	20	0.01	0.94	-	-	-
Geography	20	0.13	0.88	21	0.19	0.83
Nest stage	19	0.40	0.53	20	0.40	0.53
Laying date	18	0.53	0.48	19	0.57	0.46
Clutch	16	0.05	0.82	17	0.06	0.81
